# A nanoluciferase-expressing attenuated CHIKV vaccine strain enables rapid and visual drug screening *in vitro* and *in vivo*

**DOI:** 10.1128/jvi.00466-26

**Published:** 2026-08-11

**Authors:** Mengyuan Zheng, Zhouling Pan, Yanxian Jiang, Yan Guo, Wei Chang, Hailin Tang, Xiao Chen, Yujie Xiang, Xue Wang, Zihao Wang, Ranran Chang, Li Liu, Ping Zhao, Yue Feng, Xueshan Xia

**Affiliations:** 1 Faculty of Life Science and Technology & Yunnan Provincial Key Laboratory of Public Health and Biosafety, Kunming University of Science and Technology 47910, Kunming, Yunnan, China; 2 Department of Microbiology, Faculty of Naval Medicine, Naval Medical University 12521 , Shanghai, China; 3 Yunnan Provincial Key Laboratory of Public Health and Biosafety, Kunming Medical University 71240, Kunming, Yunnan, China; University of Kentucky College of Medicine, Lexington, Kentucky, USA

**Keywords:** chikungunya virus, VLA1553, nano-luciferase, reporter virus, drug screening, 4'-fluorouridine

## Abstract

Climate change has expanded the range of vectors for the chikungunya virus (CHIKV), thereby increasing its global threat. This situation underscores the urgent need for antiviral therapies. However, the development of drugs is limited by the need for Biosafety Level 3 (BSL-3) containment and the lack of tools for real-time infection monitoring. To address these challenges, we developed a replication-competent reporter virus called CHIKV-ΔnsP3-Nluc. We inserted the Nano-luciferase (Nluc) gene into the attenuation site of the FDA-approved live-attenuated vaccine strain VLA1553 (IXCHIQ). This modified virus retains an attenuated phenotype, replicating more slowly than wild-type CHIKV while maintaining high genetic stability. Nluc activity correlates well with viral titer (*R*^2^ = 0.9689), enabling precise quantification of viral replication. In interferon-deficient A6 mice, CHIKV-ΔnsP3-Nluc caused a lethal yet attenuated infection. Real-time bioluminescence imaging revealed that the spleen and kidneys were the primary replication sites, with luminescence intensity closely matching viral titers (*R*^2^ = 0.8388). We confirmed the platform’s potential for rapid antiviral screening using the nucleoside analog 4′-fluorouridine (4′-FlU). This compound inhibited viral replication *in vitro* (EC_50_= 0.063 µM) and significantly reduced viremia and mortality *in vivo* at a dose of 10 mg/kg, resulting in 100% survival. CHIKV-ΔnsP3-Nluc is a BSL-2-compliant tool that combines the safety of a vaccine candidate with a sensitive reporter function. It has the potential to accelerate studies of CHIKV pathogenesis and assist in developing new antiviral therapies.

**IMPORTANCE:**

The expanding range of CHIKV outbreaks poses a threat to global public health, and there is currently a lack of suitable tools for the development of antiviral drugs. In this study, we developed a Nano-luciferase reporter virus based on the attenuated vaccine strain VLA1553 to track viral infection. This viral assay can effectively evaluate the inhibitory and protective effects of candidate compounds both *in vitro* and *in vivo*, and its luciferase signal reliably reflects the level of viral replication. Our research provides a safe and stable tool that serves as a suitable platform for the development of antiviral drugs targeting CHIKV.

## INTRODUCTION

Chikungunya virus (CHIKV) is primarily transmitted by the *Aedes albopictus* and *Aedes aegypti* mosquitoes and has disseminated expeditiously on a global scale, thereby becoming a substantial public health concern (1). Infection with CHIKV results in chikungunya fever (CHIKF), a condition characterized by a sudden rise in body temperature and severe joint pain that can persist for months or even years (2). In severe cases, the condition may result in neurological complications or death. The geographic spread of mosquito vectors, in conjunction with viral mutations, has resulted in the formation of a complex epidemiological landscape (3, 4). To date, 119 countries have reported cases of transmission, placing approximately 5.5 million people at risk. In 2025, there were multiple simultaneous outbreaks, including 27 indigenous transmission events in Europe, which marked an unprecedented high. From January to June of this year, Brazil witnessed a succession of epidemics, culminating in a cumulative total of 181,275 suspected cases and 75,660 confirmed cases, accompanied by 92 fatalities. Concurrently, Bolivia reported 3,095 confirmed cases and one death (1, 5, 6). In China, Guangdong Province has documented its most significant local outbreak to date, with a cumulative total of 16,452 cases as of September 27 (7). Concurrently, Hong Kong reported its inaugural imported case in August and its first fatality in October (8, 9). The transmission of CHIKV poses a grave threat to global public health.

Despite the global dissemination of CHIKV, there are currently no specific antiviral treatments available (1). The clinical management of CHIKF is centered on alleviating symptoms through the administration of nonsteroidal anti-inflammatory drugs, such as sulfasalazine and hydroxychloroquine. However, the efficacy of these medications is constrained (10, 11). A substantial body of research indicates that certain compounds targeting viral proteins or host factors may be effective. However, the validation of these compounds in animal models remains to be substantiated (11). The development of antiviral drugs is a complex and costly process, and a significant challenge is creating a screening system that accurately mimics viral infection to evaluate candidate compounds (12). Consequently, the establishment of an efficient screening platform for CHIKV drug development has emerged as a critical research priority.

To further the study of CHIKV pathogenicity and facilitate the screening of antiviral drugs, there is an urgent need for tools that track viral replication and spread in real time in animal models. The CHIKV reporter viruses, which are engineered through the application of reverse genetics, exhibit the characteristics of wild-type viruses while incorporating reporter genes. Conventionally, eGFP, mCherry, and iRFP have been utilized for drug screening and antibody evaluation (13–16). Recently, Nano-luciferase (Nluc), a bioluminescent reporter enzyme, has emerged as a valuable tool due to its high luminescence intensity and minimal background autofluorescence, enhancing signal quality (17). Nluc, with a low molecular weight of 19 kD, facilitates integration into viral genomes without compromising protein function (18). However, the high infectivity of the current CHIKV reporter viruses necessitates the use of Biosafety Level 3 (BSL-3) laboratory conditions, which limit the virus’s broader application.

VLA1553 (IXCHIQ) is the inaugural live attenuated CHIKV vaccine to receive full FDA approval, having been launched in 2023 (19). Derived from the ECSA genotype La Reunion strain (LR2006-OPY1), it contains an 183-nucleotide deletion in the nsP3 gene. This deletion has been shown to inhibit viral replication and reduce virulence while maintaining growth in laboratory settings and immunogenicity. The employment of VLA1553 in research endeavors confers numerous advantages, including enhanced safety, immunological relevance, and genetic controllability (20). This advancement serves to propel studies in viral pathogenesis, vaccine optimization, and antiviral drug development.

In this study, the Nluc reporter gene was integrated into a CHIKV vaccine strain, thereby creating the recombinant virus CHIKV. This virus serves as a rapid, high-throughput drug screening platform, exhibiting genetic stability and replication kinetics similar to those of the wild-type strain. The correlation between its bioluminescence signal and viral load has been demonstrated to be positive, both *in vivo* and *in vitro*. In a mouse model, LR2006-Nluc replicated the infection characteristics of LR2006-wt with consistent kinetics. Preliminary validation experiments demonstrated that the incorporation of the nucleotide analog 4′-fluorouridine (4′-FlU) effectively inhibited the replication of the CHIKV and reduced the viral damage it caused. The CHIKV reporter virus has been determined to be safe for use in BSL-2 laboratories, facilitating high-throughput screening of anti-CHIKV drugs.

## MATERIALS AND METHODS

### Cell lines, virus, mice, and nucleoside analogs

Mosquito C6/36 cells were cultivated in RPMI medium (Invitrogen) with the addition of 10% fetal bovine serum (FBS; Thermo Scientific HyClone) at a temperature of 28°C. Conversely, all other cell types were maintained at 37°C in an atmosphere containing 5% CO_2_. BHK-21 (baby hamster kidney fibroblast) and U2OS (human osteosarcoma) cells were cultured in Dulbecco’s Modified Eagle’s Medium (DMEM; Invitrogen) with 10% FBS and 100 U/mL of penicillin–streptomycin. The vaccine strain of CHIKV is derived from the ECSA genotype, specifically the La Réunion strain LR2006-OPY1 (GenBank accession no. DQ443544), which has an 183-nucleotide deletion in the hypervariable region of the nsp3 gene, affecting amino acid positions 323–383 (CHIKV-ΔnsP3). Wild-type CHIKV (CHIKV-WT) and CHIKV-ΔnsP3-Nluc reporter viruses were generated from their infectious cDNA clones (CHIKV-ΔnsP3-Nluc). The mouse strains utilized in this study were 6- to 8-week-old type-I interferon-α/β receptor gene (Ifnar1)-deficient C57BL/6 mice, colloquially referred to as A6 mice. 4′-FlU was purchased from Bide Pharm (Shanghai, China).

### Plasmid construction, RNA transcription, and transfection

In this study, the genome sequence of the wild-type strain LR2006-OPY1 was used as a reference for the attenuated live vaccine VLA1553 (IXCHIQ) against CHIKV. The complete CHIKV genome sequence was synthesized and cloned into the pACYC plasmid, yielding the recombinant plasmid pACYC-CHIKV-LR2006 (CHIKV-WT). Utilizing this plasmid as a foundation, we amplified two segments of the CHIKV sequence that are deficient in amino acids 323–383 within the nsp3 protein through the PCR method (21). To that end, we employed seamless cloning technology to generate the pACYC-CHIKV-LR2006-ΔnsP3 (CHIKV-ΔnsP3) plasmid. Subsequently, the Nluc gene sequence was inserted at amino acid position 490 of the nsp3 protein in the wild-type strain, utilizing CHIKV-ΔnsP3 as the backbone. Consequently, the recombinant plasmid pACYC-CHIKV-LR2006-ΔnsP3-Nluc (CHIKV-ΔnsP3-Nluc) was developed (Fig. S1). All the above primer sequences are provided in Table S1. In addition, a T7 promoter was engineered at the 5′ end of the viral genome sequence to facilitate *in vitro* transcription. The three infectious clonal plasmids were linearized using restriction enzymes and subsequently transcribed into viral RNA *in vitro* using the T7 mMESSAGE mMACHINE kits (Invitrogen), following the manufacturer’s instructions. Following the transcription process, the RNA was introduced into BHK-21 cells using Lipofectamine 3000 Transfection Reagent (Invitrogen). Culture supernatants were collected 48–72 h post-transfection.

### Immunofluorescence assay

BHK-21 cells were infected with CHIKV-WT, CHIKV-ΔnsP3, and CHIKV-ΔnsP3-Nluc RNA, respectively, and then seeded onto cell coverslips. Forty-eight hours post-transfection, the cells were fixed at room temperature with a 1:1 mixture of ice-cold methanol and acetone for 20 min. After fixation, the anti-CHIKV E2 protein mouse monoclonal antibody (Sigma-Aldrich, St. Louis, MO, USA) was added and incubated at 37°C for 1 h. The samples were washed thrice with phosphate-buffered saline (PBS), followed by the addition of Alexa Fluor 488-conjugated goat anti-mouse IgG (Invitrogen) for incubation. Subsequently, the samples should undergo a series of three washes with PBS. Subsequently, 4′,6-diamidotoluidine diphenylindole (DAPI, Solarbio, Beijing, China) was added to stain the cell nuclei. In summary, the coverslip must be mounted on a slide containing a 50% glycerol solution. The process of capturing images is to be carried out using a fluorescence microscope (Leica DMI 3000B).

### Immunostaining plaque assay

BHK-21 cells, with a density of 2 × 10^5^ cells per well in a 12-well plate, were infected with 10-fold serial dilutions of CHIKV-WT, CHIKV-ΔnsP3, or CHIKV-ΔnsP3-Nluc viruses for a duration of 1 h at a temperature of 37°C. After the incubation period, the cells were overlaid with DMEM containing 2% FBS and 2% methyl cellulose. Three days post-infection (d.p.i.), the cells were fixed with 4% paraformaldehyde and stained with 1% crystal violet. Following the staining procedure, the cells were meticulously rinsed with tap water, and the plaque numbers and morphologies were systematically documented.

### Viral growth kinetics

BHK-21 cells, with a density of 1 × 10^5^ cells per well in a 24-well plate, were infected at a multiplicity of infection (MOI) of 0.01. The cell culture medium was subsequently collected at predetermined time points post-infection and analyzed using a plaque assay to ascertain the viral titer.

### Luciferase assay

U2OS cells were cultivated to 80%–90% confluence in 24-well plates and subsequently infected with CHIKV-WT, CHIKV-ΔnsP3, or CHIKV-ΔnsP3-Nluc viruses. Following a 48-h incubation period at 37°C in a 5% CO_2_ atmosphere, the cells were lysed using a passive lysis buffer (Promega, Madison, MI, USA). Subsequently, luciferase activity was measured using the Nano-Glo Luciferase Assay System (Promega, Madison, MI, USA) according to the manufacturer’s instructions.

### Quantitative RT-PCR

Following the manufacturer’s instructions, viral RNA was extracted using the RNAsimple Total RNA Kit (TIANGEN, Beijing, China). The quantification of RNA was conducted with the One-Step PrimeScript RT-PCR Kit (Takara, Dalian, China), targeting the CHIKV nsp2 gene. The primers and probes used were as follows: nsp2-F (5′-AACATCTGCACYCAAGTGTACCA-3′); nsp2-R (5′-TGCGCATTTTGCCTTCGTAAT-3′); and nsp2 probe (5′-ACTGCCTGTGACYGCCATTGTGTC-3′).

### Antiviral assay of CHIKV-ΔnsP3-Nluc by 4′-FlU

The experimental design involved seeding U2OS cells in a 12-well plate and culturing them until they reached 80%–90% confluence. After achieving this confluence, the cells were infected with CHIKV-ΔnsP3-Nluc at an MOI of 0.1. The viral inoculum was prepared to contain 4′-FIU at a final concentration of 10 μM and subjected to half-log10 serial dilutions, with sterile water serving as the control. Following 1 h of adsorption at 37°C, the viral inoculum was removed and replaced with fresh complete medium containing the same concentration of 4′-FIU. The cells were then incubated at 37°C in a 5% CO_2_ environment for 24 h (22). After this period, the cell culture media were collected. Nluc activity was measured using the Nano-Glo Luciferase Assay System (Promega), and the luciferase signals were recorded with a luminometer. The infection level in the control group was set at 100%, and the infection levels in drug-treated groups were normalized to this control. The inhibition rate was calculated by subtracting each treatment group’s relative infection level from 100%. EC_50_ values were analyzed using GraphPad Prism software.

### Animal experiments

All animal experiments were conducted on 6- to 8-week-old male and female mice, which were depilated three days before infection. The virus was diluted in DMEM and administered to the mice through a combination of intradermal (i.d.) and footpad injections. The final inoculum doses were as follows: CHIKV-WT at 10^4^ PFU; CHIKV-ΔnsP3 at 10^4^ PFU; and CHIKV-ΔnsP3-Nluc at 10^3^ or 10^4^ PFU. The control mice received an equivalent volume of DMEM. To monitor disease progression, daily observations were made, including the survival status, body weight changes, footpad swelling severity, and clinical signs. The endpoint for the Kaplan-Meier survival analysis was defined as the occurrence of body weight loss exceeding 20% or death, with the initial body weight set at 100%. For the *in vivo* antiviral studies, mice were infected with 10⁴ PFU of CHIKV-ΔnsP3-Nluc. At 2 h post-infection (h.p.i.), mice were treated with 4′-FIU (5 mg/kg/day or 10 mg/kg/day) or a vehicle control (10% DMSO, 5% Tween-80, 40% PEG300, and 45% saline) once daily (q.d.) until the end of the experiment (22). Whole blood samples were collected via distal tail vein puncture at predetermined time points for viremia detection. For *in vivo* imaging and subsequent histopathological analysis, mice underwent bioluminescence imaging (BLI) under anesthesia, followed by tissue collection at 1, 2, and 3 d.p.i. The harvested tissues comprised the brain, heart, spleen, kidneys, liver, skin, bilateral muscles, and bilateral footpads. *Ex vivo* tissues were divided into two portions after imaging: one portion was stored at −80°C for subsequent experimental analysis, while the other was fixed and embedded in paraffin for histopathological examination (23).

### Bioluminescence imaging

The mice utilized in this study were anesthetized through intraperitoneal (i.p.) injection of a 1.25% solution of 2,2,2-tribromoethanol (AibeiBio, Nanjing, China). The Nano-Glo Fluorofurimazine (Promega) was prepared according to the manufacturer’s instructions and subsequently diluted with PBS to a 1:5 ratio. Subsequently, a 100-μL intraperitoneal injection of the diluted substrate was administered to each mouse. The imaging process was conducted using the IVIS Lumina LT system (PerkinElmer), a specialized instrument designed for such applications. Images of live animals were acquired under standardized exposure conditions with an exposure time of 40 s. Following tissue collection, excised organs were subjected to *ex vivo* bioluminescence imaging with an exposure time of 60 s. All images were analyzed using Living Image software, version 4.4, developed by Caliper Life Sciences. The quantification of bioluminescence signals was achieved by delineating regions of interest (ROIs) on images that had been uniformly scaled. The data were expressed as total photon flux, defined as photons per second (p/s) (23).

### Titration of virus from excised organs

The excised organs were weighed and homogenized using zirconia beads (Novastar) in 500 μL of DMEM. Subsequently, the homogenates were subjected to a centrifugation process at 3,000 revolutions per minute for 10 min to collect the viral supernatant. The viral titer was determined using the immunostaining plaque assay previously described.

### Histopathology analysis

Tissues were fixed with 4% paraformaldehyde in PBS and subsequently embedded in paraffin according to standard procedures. Subsequently, the embedded tissues were sectioned at a thickness of 4 μm for staining with hematoxylin and eosin. The histopathological evaluation of tissues was performed on hematoxylin and eosin (H&E)-stained sections by two independent observers who were blinded to the experimental groups. The lesions evaluated in each tissue, along with their corresponding weighting factors, are comprehensively outlined in Table S2. The weighting factors were assigned according to the pathological significance of each lesion. Irreversible tissue damage or lesions associated with major functional impairment, such as necrosis in skin and paw tissues and white pulp reduction in spleen tissue, were assigned higher weights, whereas reversible changes such as edema were assigned lower weights. The severity of each lesion was assessed in five representative microscopic fields and assigned a lesion score (LS) ranging from 0 to 4, where 0 = normal tissue, 1 = very mild, 2 = mild, 3 = moderate, and 4 = severe lesion. The tissue histopathological score (HS) was calculated using the following formula: HS = Σ (LS × WF).

### Statistics analysis

The statistical significance of the observed differences in survival rates was determined using a Kaplan-Meier analysis, conducted with Prism version 4.00 for Windows (GraphPad Prism 10). All titration data were subjected to log-transformation and subsequently compared using an unpaired Student’s *t*-test. The correlation between PFU and fluorescence values was determined through the analysis of the relevant curves using Pearson’s correlation coefficient and a 95% confidence interval.

## RESULTS

### Development and characterization of the stable reporter CHIKV-ΔnsP3-Nluc strain

**Fig 1:**
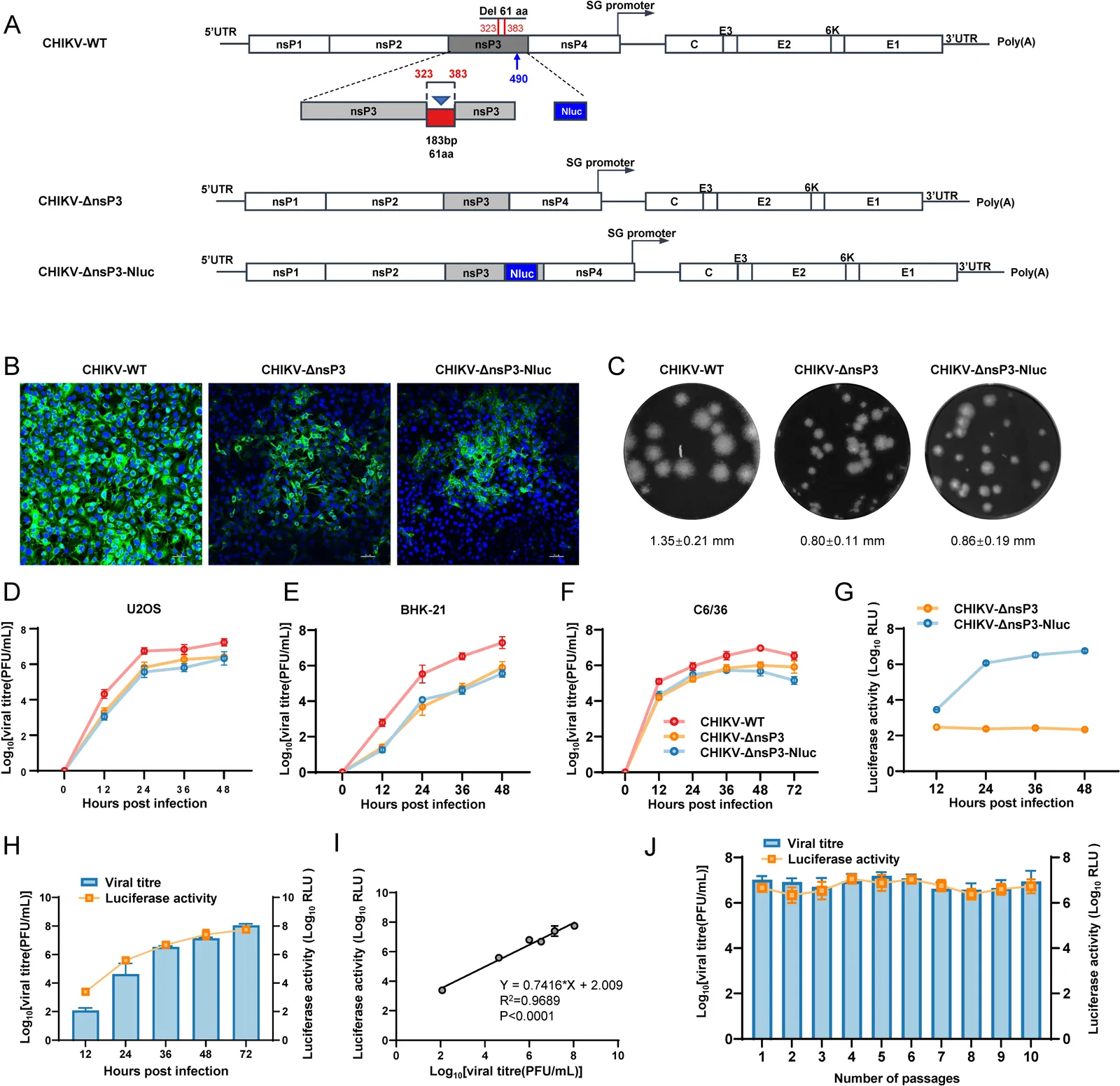
Construction and characterization of the stable reporter CHIKV-ΔnsP3-Nluc. (**A**) Schematic representation of CHIKV-WT, CHIKV-ΔnsP3, and CHIKV-ΔnsP3-Nluc reporter viruses. An infectious cDNA clone of CHIKV was used as a backbone for the construction of the CHIKV reporter virus. The expression of the Nano luminescence (Nluc) reporter gene was inserted at amino acid position 490 of the nsp3 protein in the wild-type strain. (**B**) Immunofluorescence assay (IFA) analysis of viral E2 expression in BHK-21 cells transfected with *in vitro* transcribed genome-length RNAs of CHIKV-WT, CHIKV-ΔnsP3, and CHIKV-ΔnsP3-Nluc. (**C**) Plaque morphology of CHIKV-WT, CHIKV-ΔnsP3, and CHIKV-ΔnsP3-Nluc reporter viruses in BHK-21 cells. (**D–F**) Comparison of the growth kinetics of CHIKV-WT, CHIKV-ΔnsP3, and CHIKV-ΔnsP3-Nluc reporter viruses was conducted at an MOI of 0.01 using three cell lines: U2OS, BHK-21, and C6/36 (*n* = 3, error bars indicate SD from triplicates). (**G**) Replication kinetics of CHIKV-ΔnsP3 and CHIKV-ΔnsP3-Nluc in U2OS cells infected with an MOI of 0.01 were determined by luciferase activity measurements of the cell lysates (*n* = 3, error bars indicate SD from triplicates). (**H**) Correlation between viral titers and luciferase activity of CHIKV-ΔnsP3-Nluc during *in vitro* replication kinetics. (**I**) Linear correlation between viral titers and luciferase activities of CHIKV-ΔnsP3-Nluc *in vitro*. The Pearson correlation coefficient and two-tailed *P* value obtained through a linear regression analysis are shown (*n* = 3, error bars indicate SD from triplicates). (**J**) Stability of the recombinant CHIKV-ΔnsP3-Nluc. BHK-21 cells were serially infected for 10 passages with CHIKV-ΔnsP3-Nluc to assess the stability of the virus. At each passage, the viral titer of the supernatant was measured (curve), as well as the Nluc activity (bars), expressed as relative Nluc in the infected cells. Curves and histograms show the median value and interquartile range of measurements from three replicates.

To create the recombinant CHIKV-ΔnsP3-Nluc, the bioluminescent gene Nluc was strategically integrated into the full-length infectious cDNA clone of CHIKV-ΔnsP3 (Fig. 1A).

Equal amounts of CHIKV-WT, CHIKV-ΔnsP3, and CHIKV-ΔnsP3-Nluc genomic RNAs, transcribed from infectious cDNA clones, were used to transfect BHK-21 cells. The assessment of the replication capabilities of these viruses was conducted using an IFA with an anti-E2 mouse polyclonal antibody (Fig. 1B). The results of the IFA test indicated the presence of IFA-positive cells in all three RNA-transfected cell lines. However, the number of IFA-positive cells in the CHIKV-ΔnsP3 and CHIKV-ΔnsP3-Nluc groups was similar but lower than in the WT-CHIKV group. Consistent with IFA results, the plaques formed by CHIKV-ΔnsP3 and CHIKV-ΔnsP3-Nluc exhibited comparable dimensions; however, they were substantially smaller in size when compared to those produced by CHIKV-WT (Fig. 1C). The three strains exhibited effective replication in the U2OS, BHK-21, and C6/36 cell lines. However, the replication rates of CHIKV-ΔnsP3 and CHIKV-ΔnsP3-Nluc were similar and notably slower than the replication rate of WT-CHIKV (Fig. 1D through F). Overall, the data suggest that CHIKV-ΔnsP3 and CHIKV-ΔnsP3-Nluc demonstrate consistent replication and infectivity capabilities, though with reduced viral efficiency compared to CHIKV-WT.

The growth kinetics of the CHIKV-ΔnsP3-Nluc virus, as determined through luciferase activity assays, suggest that this virus possesses a high replication capacity in U2OS cells (Fig. 1G). Luciferase activity exhibited a robust correlation with alterations in viral titer (Fig. 1H). Furthermore, a strong linear correlation (*R*² =0.9689) was demonstrated between luciferase activities and the viral titers of CHIKV-ΔnsP3-Nluc (Fig. 1I). Furthermore, to assess the genetic stability of the CHIKV-ΔnsP3-Nluc strain, the recombinant virus was subjected to 10 consecutive passages in BHK-21 cells (2–3 days per passage). The virus titer and Nluc fluorescence activity were measured for samples collected from passages P1 to P10. The findings demonstrated that, under conditions of equivalent viral titer, Nluc activity exhibited consistent and stable levels throughout all passage generations (Fig. 1J), indicating the robust stability of CHIKV-ΔnsP3-Nluc during serial passage.

### 4′-FlU anti-CHIKV activity assay using CHIKV-ΔnsP3-Nluc strain *in vitro*

**Fig 2:**
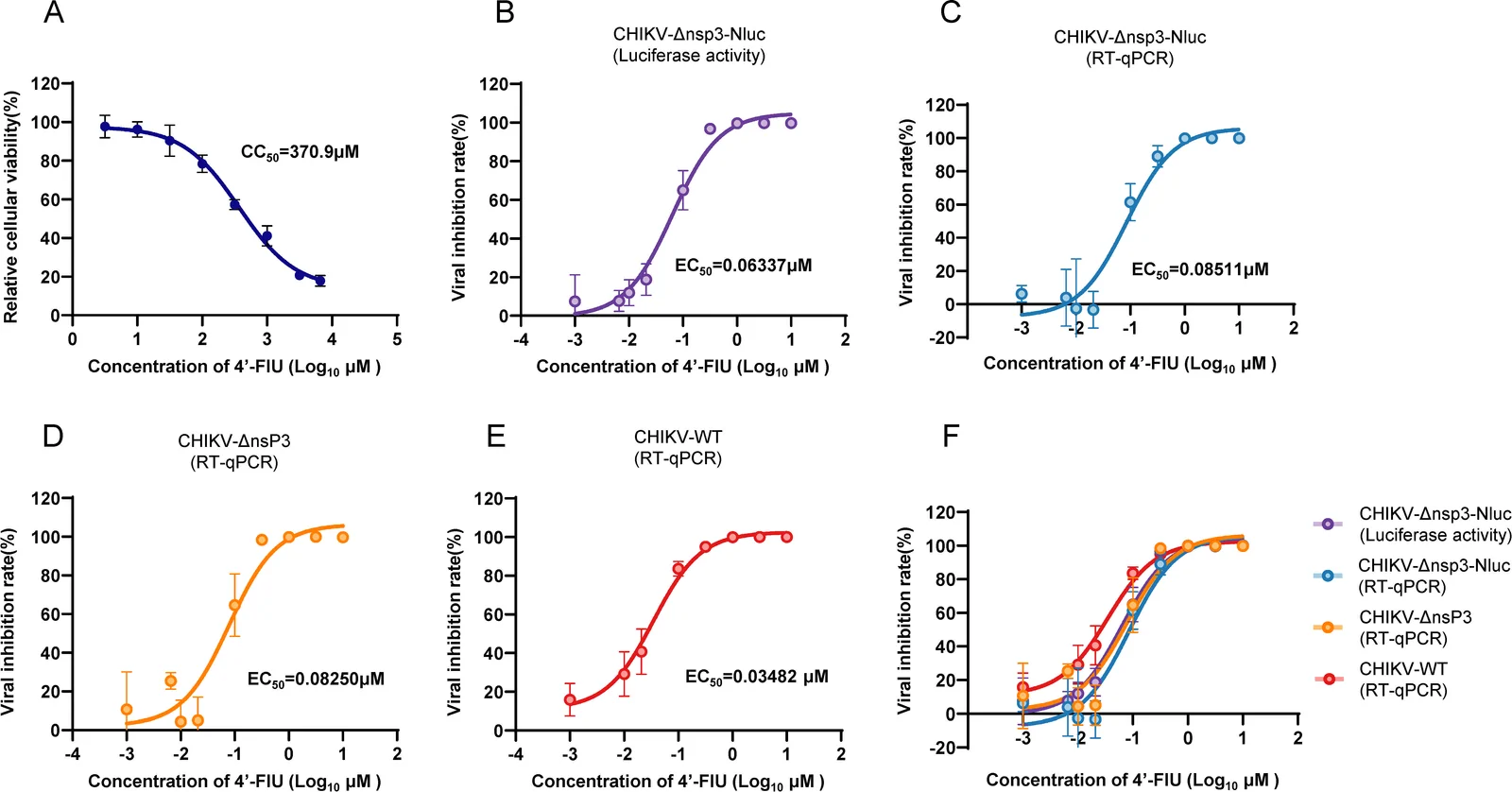
Antiviral activity of 4′-FlU on CHIKV-ΔnsP3-Nluc reporter viruses. (**A**) Assessment of the cytotoxicity of 4′-FlU at different doses on U2OS cells. (**B**) Dose-dependent effects of 4′-FlU on CHIKV-ΔnsP3-Nluc replication in U2OS cells. U2OS cells pretreated with a series of concentrations of 4′-FlU or DMSO were infected with CHIKV-ΔnsP3-Nluc (MOI = 0.1). The cells were lysed, and luciferase activities were measured at 24 h.p.i. (*n* = 3, error bars indicate SD from triplicates). (**C**) Dose-dependent effects of 4′-FlU on CHIKV-ΔnsP3-Nluc replication in U2OS cells as determined by qRT-PCR (*n* = 3, error bars indicate SD from triplicates). (**D**) Dose-dependent effects of 4′-FlU on CHIKV-ΔnsP3 replication in U2OS cells as determined by qRT-PCR (*n* = 3, error bars indicate SD from triplicates). (**E**) Dose-dependent effects of 4′-FlU on CHIKV-WT replication in U2OS cells as determined by qRT-PCR (*n* = 3, error bars indicate SD from triplicates). (**F**) Integration of the detection curves from panels B to E.

Recent studies have demonstrated the capacity of 4′-FlU to impede the replication of CHIKV. To further substantiate the correlation between Nluc fluorescence activity and viral replication, the antiviral efficacy of 4′-FlU was evaluated using the CHIKV-ΔnsP3-Nluc reporter virus. The half-maximal cytotoxic concentration (CC_50_) and 50% effective concentration (EC_50_) of 4′-FlU in U2OS cells infected with CHIKV-ΔnsP3-Nluc were found to be 370.9 μM and 0.06337 μM, respectively (Fig. 2A and B). Furthermore, analogous EC_50_ values were ascertained for 4′-FlU with CHIKV-ΔnsP3-Nluc (EC_50_ = 0.08511 µM), CHIKV-ΔnsP3 (EC_50_ = 0.08250 µM), and CHIKV-WT (EC_50_ = 0.03482 µM), as determined by the quantitation of viral RNA copies (Fig. 2C through F). These findings further confirm that Nluc fluorescence activity can serve as a reliable surrogate for the replication of CHIKV-WT. Additionally, this study suggests that the CHIKV-ΔnsP3-Nluc reporter virus system can be utilized for screening potential anti-CHIKV inhibitors.

### Establishment of a visualized animal model of CHIKV-ΔnsP3-Nluc strain

**Fig 3:**
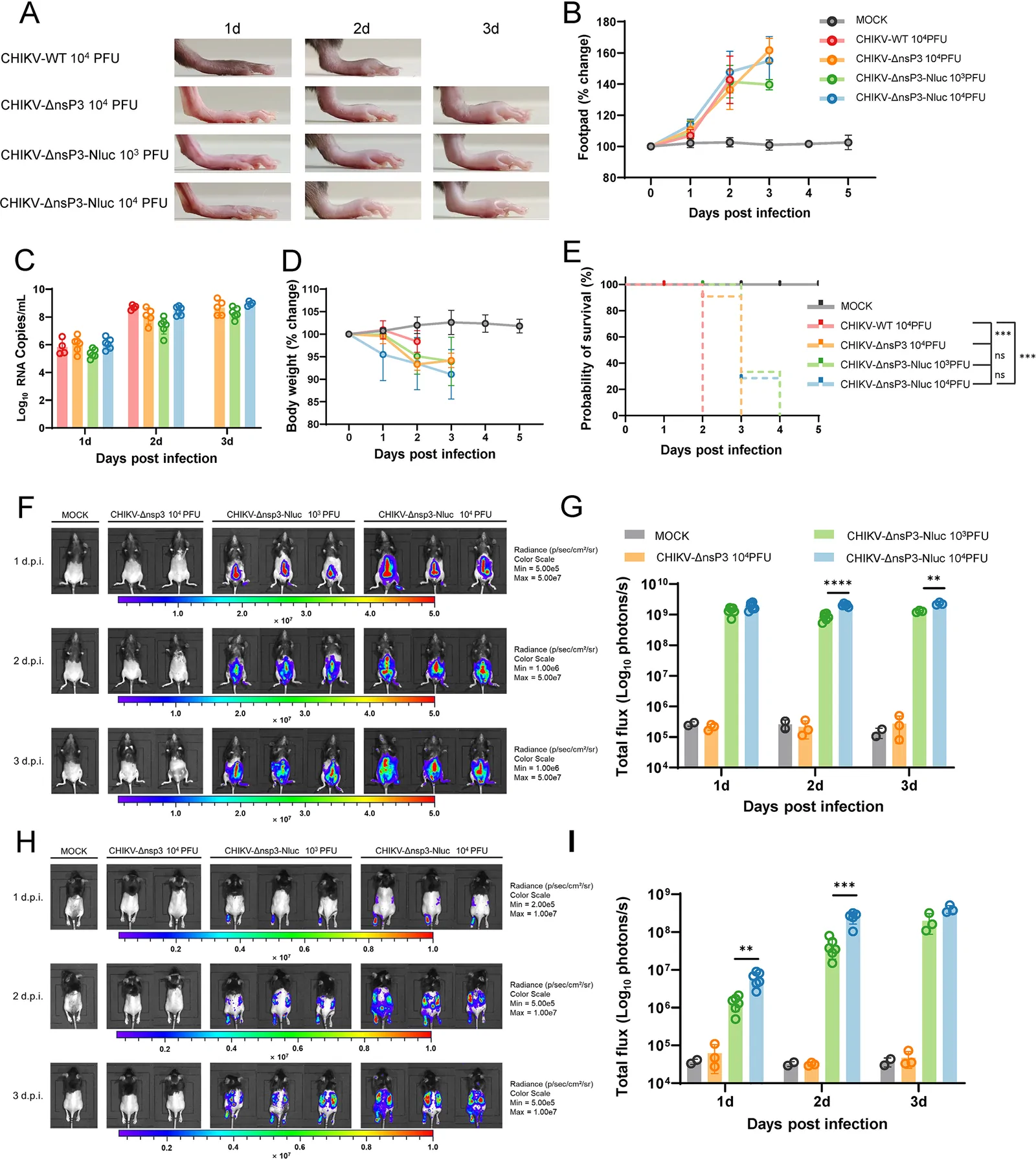
Characterization of CHIKV-ΔnsP3-Nluc infection in A6 mice. (**A**) The changes in swelling of the ipsilateral paw within 3 days after inoculation in mice infected with the three strains: CHIKV-WT, CHIKV-ΔnsP3, and CHIKV-ΔnsP3-Nluc. (**B**) Swelling of the same side foot was measured within 3 days after vaccination using digital calipers. (**C**) Changes in viremia were observed within 3 days post-inoculation of mice with three viral strains: CHIKV-WT, CHIKV-ΔnsP3, and CHIKV-ΔnsP3-Nluc (*n* = 4–6 per group, each dot represents an individual mouse). (**D**) Temporal changes in mouse body weight; initial body weight was set to 100% (*n* = 4–6 per group). (**E**) Kaplan-Meier survival curve of A6 mice infected with the indicated viruses as compared by the log-rank (Mantel-Cox) test (*n* = 6 per group). (**F**) Representative images illustrating noninvasive BLI of CHIKV-ΔnsP3-Nluc-infected mice in the ventral position at 1, 2, and 3 d.p.i. Images of one mock-infected mouse and CHIKV-ΔnsP3-infected mice were included as controls. The color bar, ranging from blue to red, represents the computed signal intensities. (**G**) Temporal quantification of bioluminescence imaging data was calculated as the photon flux (photons/s) for the ventral sides of the whole body, corresponding to panel F. (**H**) Representative images illustrating noninvasive BLI of CHIKV-ΔnsP3-Nluc-infected mice in the dorsal position at 1, 2, and 3 d.p.i. Images of one mock-infected mouse and CHIKV-WT-infected mice were included as controls. The color bar, ranging from blue to red, represents the computed signal intensities. (**I**) Temporal quantification of bioluminescence imaging data was calculated as the photon flux (photons/s) for the dorsal sides of the whole body, corresponding to panel H. In panels G and I, each dot represents an individual mouse. At 1–3 d.p.i., the MOCK and CHIKV-ΔnsP3 groups consisted of 2 and 3 mice, respectively. The CHIKV-ΔnsP3-Nluc group contained 6 mice at 1–2 d.p.i.; 3 mice were sacrificed for tissue collection at 2 d.p.i., and 3 mice remained at 3 d.p.i.

This study aims to clarify the differences in disease characteristics among CHIKV-WT, CHIKV-ΔnsP3, and CHIKV-ΔnsP3-Nluc in A6 mice. The primary question is whether Nluc activity *in vivo* correlates with the progression of the disease observed with CHIKV-WT. Six- to eight-week-old A6 mice were divided into four groups and infected via intradermal and footpad routes with the following doses: 10^4^ PFU of CHIKV-WT, 10⁴ PFU of CHIKV-ΔnsP3, 10³ PFU of CHIKV-ΔnsP3-Nluc, and 10⁴ PFU of CHIKV-ΔnsP3-Nluc. Infection characteristics and Nluc activity were monitored over time. The results showed that all infected groups exhibited paw swelling beginning on day 1 post-infection, reaching 1.5–1.6 times baseline by day 3 (Fig. 3A and B). Viremia levels increased consistently in all groups between days 1 and 3 (Fig. 3C), accompanied by significant weight loss (Fig. 3D). A sequential occurrence of fatalities was observed among all infected groups. Specifically, all mice in the CHIKV-WT group died by day 2, while no deaths were observed in the CHIKV-ΔnsP3-Nluc group until day 4. Statistical analysis confirmed that infection with the CHIKV-ΔnsP3-Nluc strain resulted in a significantly delayed mortality rate compared to infection with the wild-type strain (Fig. 3E).

To further validate the tracing capability of *in vivo* Nluc activity, this study employed a small animal imaging system to dynamically monitor Nluc bioluminescence signals in A6 mice after viral infection. The results demonstrated that the bioluminescence signal disseminated rapidly from the injection site, reaching its maximum on day 3 post-infection (Fig. 3F and H). In contrast, no bioluminescence was detected in CHIKV-ΔnsP3 or mock-infected mice treated with the same Nano-Glo reagent. Furthermore, the group infected with 10⁴ PFU exhibited significantly stronger luminescence signals in both the ventral and dorsal regions compared to the group infected with 10³ PFU of CHIKV-ΔnsP3-Nluc (Fig. 3F and H). The study also employed noninvasive imaging technology to quantitatively analyze the temporal dynamics of photon flux from the luminescent signals on the ventral and dorsal sides of the mice. This methodological framework enabled the precise quantification of *in vivo* Nluc activity characteristics (Fig. 3G and I).

**Fig 4:**
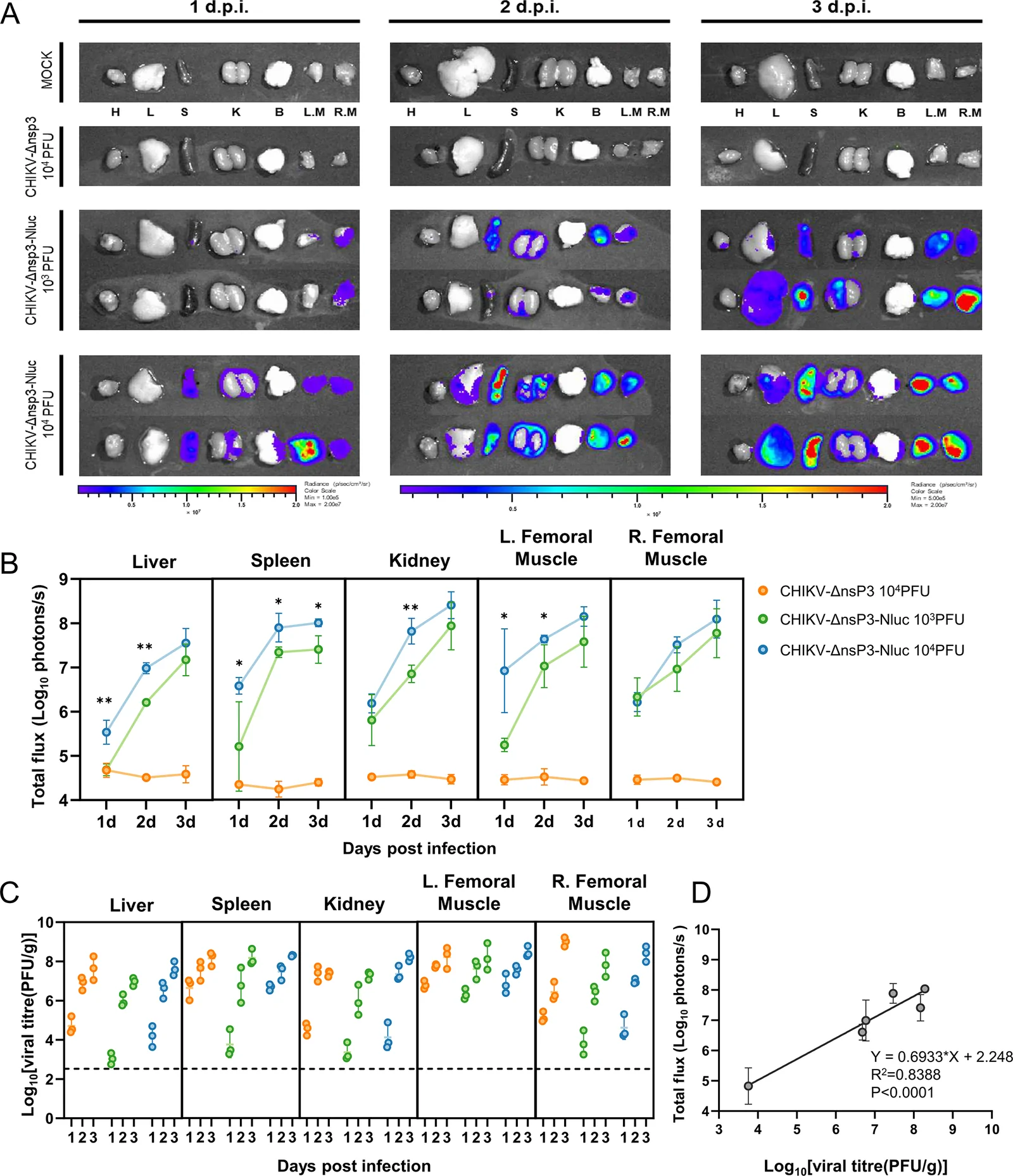
Dissemination and tissue localization of CHIKV-ΔnsP3-Nluc in A6 mice. (**A**) Representative images of *ex vivo* bioluminescence imaging of the specified organs and tissues collected from mice infected with 10^3^ PFU of CHIKV-ΔnsP3-Nluc and 10^4^ PFU of CHIKV-ΔnsP3-Nluc at 1, 2, and 3 days d.p.i. Following necropsy, representative isolated organs, including the heart (H), liver (L), spleen (S), kidney (K), brain (B), left femoral muscle (L.M), and right femoral muscle (R.M). BLI results from organs and tissues of mock-infected mice are also presented. (**B**) The replication of CHIKV-ΔnsP3-Nluc in the indicated organs of each mouse as determined by quantification of bioluminescence signal, calculated as photon flux (photons/s) at 1, 2, and 3 d.p.i., corresponding to panel A. (**C**) Viral titers in the specified organs from mice infected with 10^4^ PFU of CHIKV-ΔnsP3, 10^3^ PFU of CHIKV-ΔnsP3-Nluc, and 10^4^ PFU of CHIKV-ΔnsP3-Nluc. Mice were euthanized at 1, 2, and 3 d.p.i. (*n* = 3; error bars represent standard deviation). (**D**) Linear correlation between viral titers and bioluminescence signals in the spleen *in vivo*. The Pearson correlation coefficient and two-tailed *P* value, obtained through linear regression analysis, are presented (*n* = 3; error bars represent standard deviation).

To determine the target organs of the Nluc-tagged CHIKV-ΔnsP3-Nluc strain in mice. To this end, bioluminescence imaging technology was employed to detect bioluminescent signals in *ex vivo* organs from mice dissected on days 1, 2, and 3 post-infection. The results demonstrated sustained and significant bioluminescent signals in the liver, spleen, kidneys, and muscle tissues of the mice, with signal intensity increasing over time and peaking on day 3 post-infection (Fig. 4A). In contrast, the signals in brain and heart tissues were found to be markedly weaker and almost undetectable. Subsequent quantitative analysis of the *ex vivo* organ bioluminescence signals established the following ranking of susceptibility for CHIKV-ΔnsP3-Nluc: spleen, kidneys, muscle, and liver (Fig. 4B). Furthermore, the detection of viral titers indicated consistent replication profiles between the CHIKV-ΔnsP3 and CHIKV-ΔnsP3-Nluc strains in susceptible organs during days 1–3 post-infection. In the spleen and kidneys of mice infected with 10⁴ PFU of CHIKV-ΔnsP3-Nluc, there was no significant difference compared to the 10³ PFU infection group regarding viral titers (Fig. 4C). Furthermore, correlation analysis confirmed a significant positive relationship between viral titers in the spleens of CHIKV-ΔnsP3-Nluc-infected mice and bioluminescence signal intensity (*R*² =0.8388), indicating that the intensity of the bioluminescence imaging signal is directly reflective of viral replication levels *in vivo* (Fig. 4D).

### 4'-FlU anti-CHIKV activity assay using CHIKV-ΔnsP3-Nluc strain *in vivo*

Before evaluating the antiviral efficacy of 4′-FlU in CHIKV-ΔnsP3-Nluc-infected mice, we first assessed the *in vivo* tolerability of 4′-FlU. A6 mice were orally administered 4′-FlU at doses of 5 mg/kg, 10 mg/kg, or 20 mg/kg, and body weight changes were monitored over a 7-day observation period. The results showed that 5 mg/kg and 10 mg/kg 4′-FlU were better tolerated, whereas 20 mg/kg 4′-FlU caused a more pronounced decrease in body weight (Fig. S3). Based on these results, 5 mg/kg and 10 mg/kg were selected for subsequent *in vivo* antiviral efficacy evaluation.

**Fig 5:**
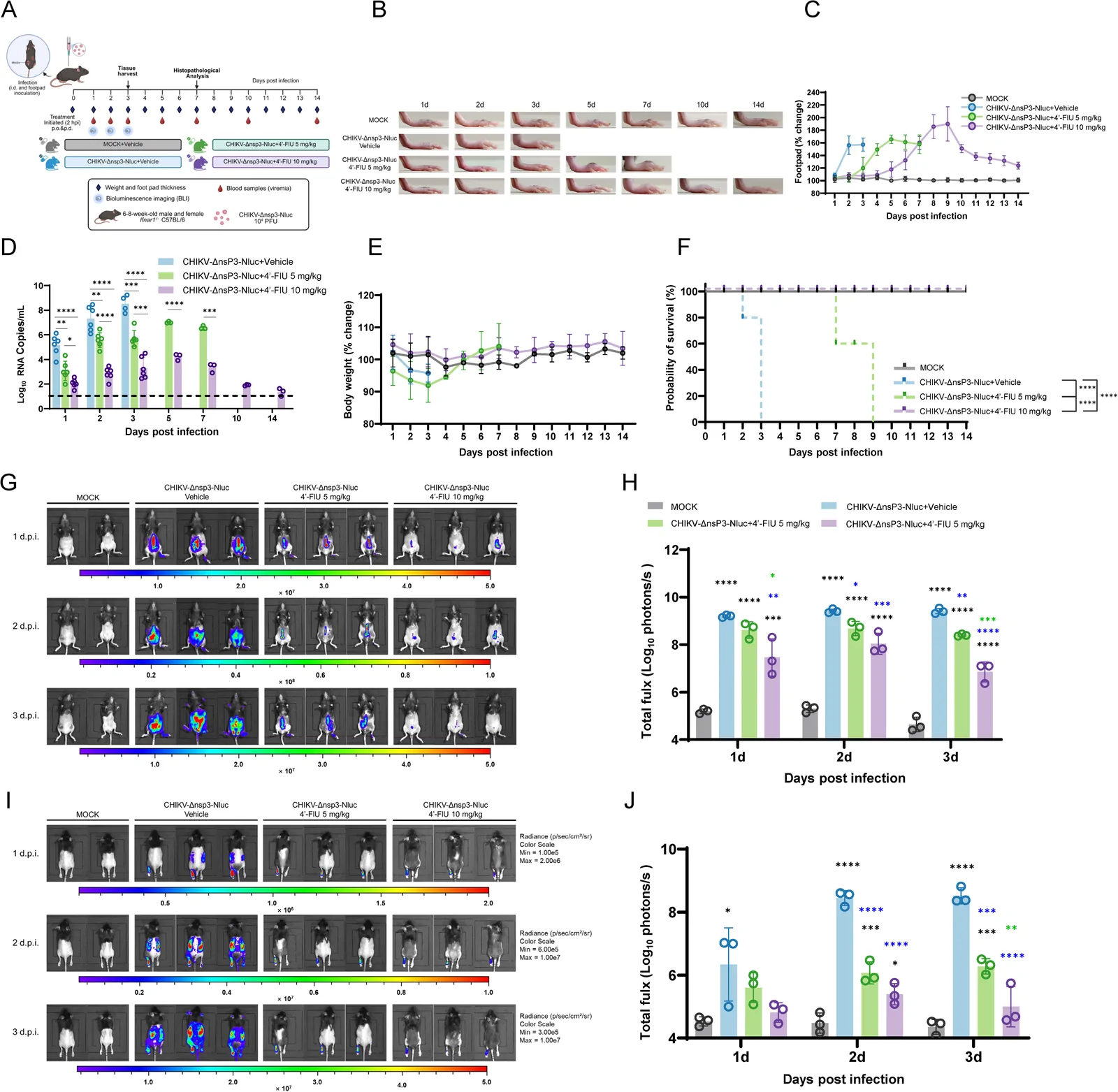
Evaluation of 4′-FIU based on CHIKV-ΔnsP3-Nluc in A6 mice. (**A**) Title: Schematic of experimental design. A6 mice were inoculated with 10^4^ PFU of CHIKV-ΔnsP3-Nluc in the left rear footpad. Two hours after inoculation, the mice were treated orally with either a vehicle control, 5 mg/kg, or 10 mg/kg of 4′-FIU. Treatment continued once daily until the predetermined experimental endpoints were reached. (**B**) Paw swelling in mice infected with CHIKV-ΔnsP3-Nluc within 14 days of receiving 5 mg/kg and 10 mg/kg 4′-FlU drug treatment. (**C**) The swelling of the ipsilateral foot over time was measured using digital calipers. (**D**) Changes in viral load were observed in mice infected with the CHIKV-ΔnsP3-Nluc viral strain after treatment with vehicle control, 5 mg/kg, or 10 mg/kg of 4′-FIU over 14 days. (**E**) Temporal changes in mouse body weight; initial body weight was set to 100% (*n* = 6 per group). (**F**) Kaplan-Meier survival curve of A6 mice infected with the indicated viruses, as compared by the log-rank (Mantel-Cox) test (*n* = 6 per group). (**G**) Representative images from noninvasive bioluminescence imaging of CHIKV-ΔnsP3-Nluc-infected mice treated with a vehicle control, 5 mg/kg, or 10 mg/kg of 4′-FIU in the ventral position at 1, 2, and 3 d.p.i. (**H**) The temporal quantification of bioluminescence imaging data was calculated as photon flux (photons/s) for the ventral sides of the entire body, as shown in panel G. Statistical significance was determined using Student’s *t*-test, with *P < 0.05, **P < 0.01, and ***P < 0.001. (**I**) Representative images from noninvasive BLI of CHIKV-ΔnsP3-Nluc-infected mice treated with a vehicle control, 5 mg/kg, or 10 mg/kg of 4′-FIU in the dorsal position at 1, 2, and 3 d.p.i. (**J**) The temporal quantification of bioluminescence imaging data was calculated as photon flux (photons/s) for the dorsal sides of the entire body, as shown in panel I. Statistical significance was determined using Student’s *t*-test, with *P < 0.05, **P < 0.01, and ***P < 0.001.

To evaluate the antiviral activity of 4′-FlU against CHIKV, in this study, A6 mice were infected with the CHIKV-ΔnsP3-Nluc strain at a dose of 10⁴ PFU. The experimental protocol and detection workflow are illustrated in Fig. 5A. The results demonstrated that paw swelling in the vehicle control group exhibited a rapid progression after infection, reaching its peak on day 3. In the group treated with 5 mg/kg of 4′-FlU, the peak swelling was delayed until day 5, while the 10 mg/kg 4′-FlU group exhibited a further delay, with peak swelling occurring on day 9. These findings suggest that 4′-FlU significantly impedes the progression of paw swelling in mice (Fig. 5B and C). Viremia detection revealed a significant reduction in CHIKV viral loads in all 4′-FlU-treated groups when compared to the vehicle control group. The 10 mg/kg 4′-FlU group exhibited the most substantial decrease in blood viral copy numbers (Fig. 5D). With respect to body weight and survival, both the vehicle control and the 5 mg/kg 4′-FlU-treated mice exhibited significant weight loss starting on day 2 post-infection (Fig. 5E). The mortality rate of the mice in the vehicle control group was observed to be complete by day 3. In contrast, the mice treated with 5 mg/kg 4′-FlU demonstrated a recovery in weight beginning on day 5, yet succumbed within 7 days. Conversely, the infected mice in the 10 mg/kg 4′-FlU group exhibited stable body weight throughout the study and survived (Fig. 5E and F). Furthermore, the results of the bioluminescence imaging further confirmed that bioluminescence signals were significantly attenuated in all 4′-FlU-treated groups compared to the solvent control group. Mice in the 10 mg/kg 4′-FlU group exhibited only faint signals in both the ventral and dorsal regions (Fig. 5G and I). Quantitative analysis of the photon flux from the ventral and dorsal bioluminescence signals in the mice corroborated these qualitative observations, demonstrating significantly lower signal intensity in the 10 mg/kg 4′-FlU group compared to the other two groups (Fig. 5H and J).

**Fig 6:**
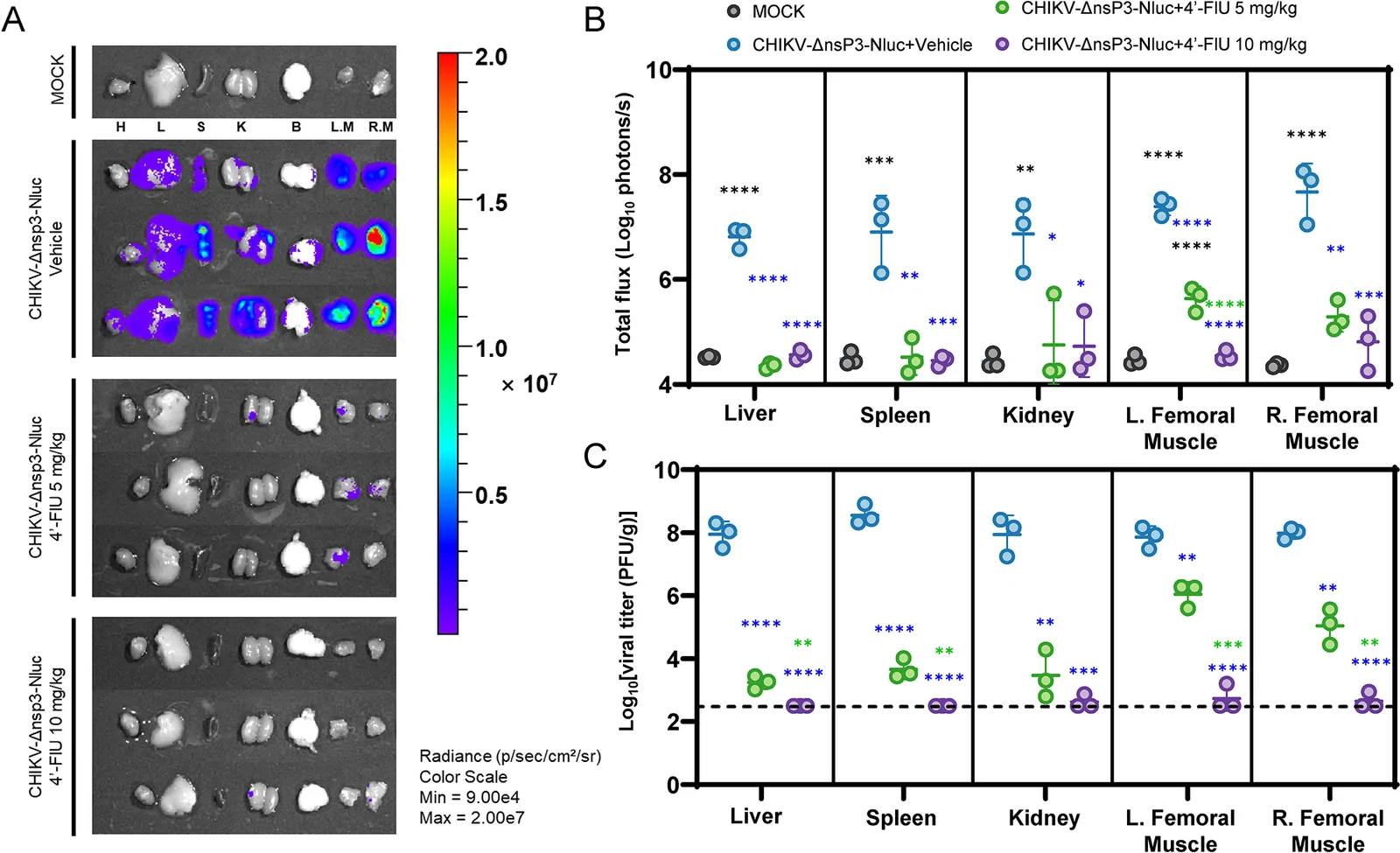
Antiviral efficacy of 4′-FIU in various tissues of A6 mice infected with CHIKV-ΔnsP3-Nluc. (**A**) Representative images of *ex vivo* bioluminescence imaging of the specified organs and tissues collected from CHIKV-ΔnsP3-Nluc-infected mice. The mice were treated with a vehicle, 5 mg/kg, or 10 mg/kg of 4′-FIU and were euthanized at 3 d.p.i. These images are compared with those from mock-infected mice. (**B**) The replication of CHIKV-ΔnsP3-Nluc in the specified organs of each mouse (*n* = 3) was assessed by quantifying the bioluminescence signal, measured as photon flux (photons/s) at 3 d.p.i., as shown in panel A. Statistical significance was determined using a non-parametric one-way ANOVA analysis, with *P*-values indicated as follows: *P < 0.05, **P < 0.01, and ***P < 0.001. (**C**) Viral titers in the organs specified in panel A.

Subsequently, target organs were harvested from mice in each group for further analysis. *In vitro* bioluminescence imaging was conducted on heart, liver, spleen, kidney, brain, and muscle tissues from mice infected with CHIKV-ΔnsP3-Nluc and treated with varying concentrations of 4′-FlU. The results indicated that the bioluminescence signal intensity in the groups treated with 4′-FlU was significantly reduced compared to the solvent control group (Fig. 6A). The oral administration of 5 mg/kg of 4′-FlU to mice resulted in the detection of only faint signals in the kidneys and left leg muscles (Fig. 6A). A quantitative analysis of photon flux supported these qualitative observations regarding bioluminescence intensity across the organs (Fig. 6B). Furthermore, assays measuring viral titer and viral load in the organs demonstrated a strong correlation with the imaging data (Fig. 6C).

**Fig 7:**
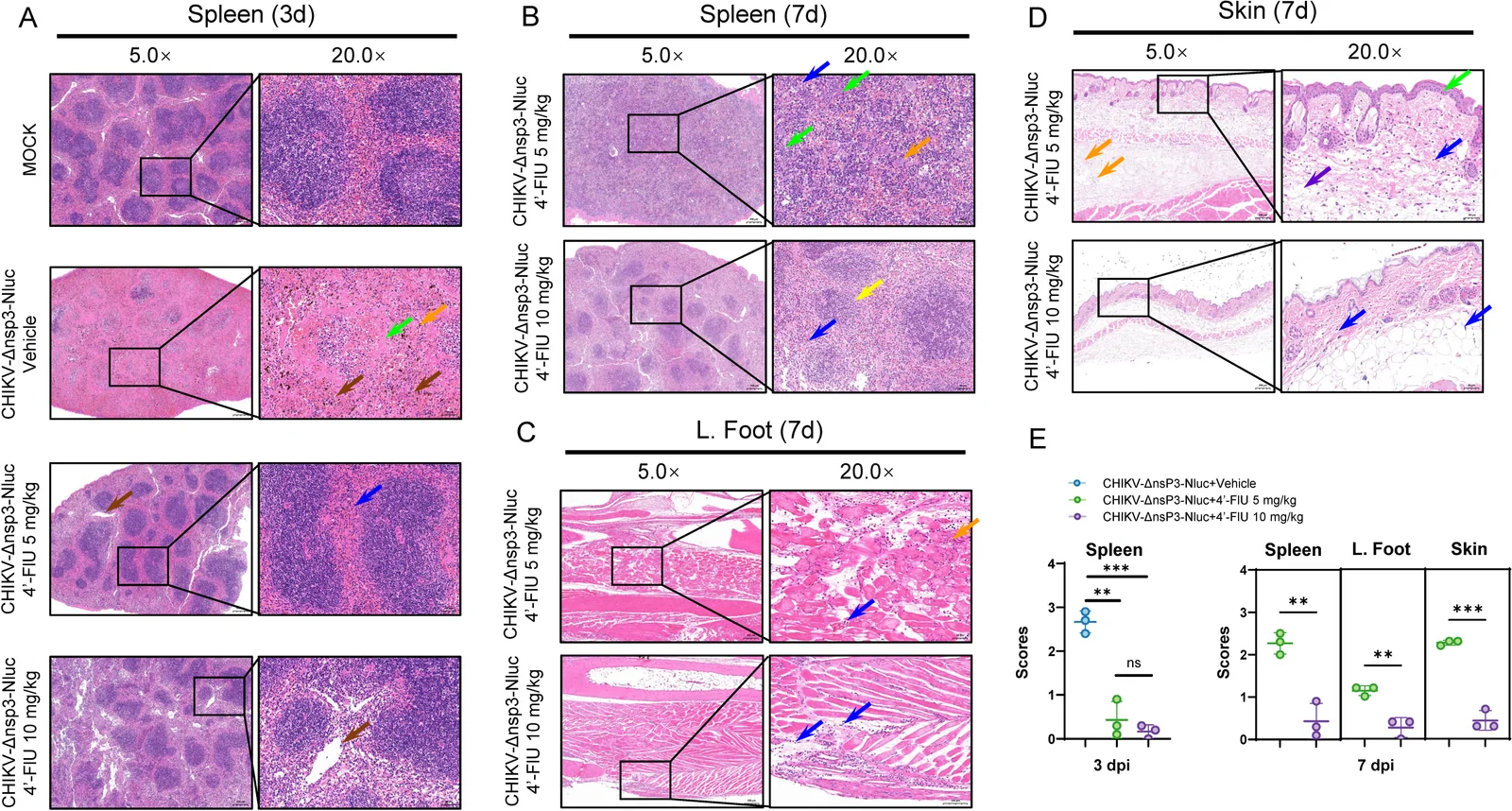
Histopathological analysis of CHIKV-ΔnsP3-Nluc-infected A6 mice after 4′-FlU treatment. (**A**) Representative H&E-stained spleen sections from mock-infected mice and CHIKV-ΔnsP3-Nluc-infected A6 mice treated with vehicle, 5 mg/kg 4′-FlU, or 10 mg/kg 4′-FlU at 3 d.p.i. 4′-FlU treatment alleviated CHIKV-ΔnsP3-Nluc-induced histopathological changes in the spleen. Scale bars represent 500 μm in the low-magnification images (×5), and boxed areas indicate the regions shown at higher magnification (×20). (**B–D**) Representative H&E-stained sections of the spleen (**B**), left foot/paw (**C**), and skin (**D**) from CHIKV-ΔnsP3-Nluc-infected A6 mice treated with vehicle, 5 mg/kg 4′-FlU, or 10 mg/kg 4′-FlU at 7 d.p.i. Representative pathological changes are indicated by color-coded arrows. In spleen sections, green arrows indicate reduction of white pulp, blue arrows indicate inflammatory cell infiltration, yellow arrows indicate extramedullary hematopoiesis, orange arrows indicate increased megakaryocytes, and brown arrows indicate red pulp congestion with sinusoidal dilatation. In paw sections, orange arrows indicate necrosis, and blue arrows indicate inflammatory cell infiltration. In skin sections, orange arrows indicate necrosis, blue arrows indicate inflammatory cell infiltration, purple arrows indicate edema, and green arrows indicate epidermal thickening. (**E**) Quantification of histopathological tissue damage scores corresponding to panels A to D.

A histopathological examination of H&E-stained tissues on Day 3 indicated that both the 5 mg/kg and 10 mg/kg treatment groups of 4′-FlU exhibited significant protective effects on spleen tissue in mice that were most susceptible to CHIKV infection, compared to the vehicle-treated group. As demonstrated in Fig. 7A, severe splenic lesions were observed in the vehicle-treated group at 3 d.p.i., indicating a reduction of white pulp (green arrows), extensive marginal zone necrosis, and mild congestion. Conversely, mice treated with 5 or 10 mg/kg exhibited significantly preserved splenic architecture, with only minor inflammatory cell infiltration (blue arrows) and sinusoidal dilatation (brown arrows). However, no significant protective effects were observed in other tissues (Fig. S4). It is noteworthy that all mice in the 5 mg/kg 4′-FlU treatment group had succumbed by Day 9 (Fig. 5F). Subsequent multi-organ histopathological analysis conducted on Day 7 revealed that the 10 mg/kg 4′-FlU group exhibited significantly greater protective effects on CHIKV-susceptible tissues (including the spleen, left foot, and skin) compared to the 5 mg/kg group (Fig. 7B through D; Fig. S5). In the spleen, the 5 mg/kg group exhibited a nearly complete loss of white pulp architecture (Fig. 7B, green arrows), while the 10 mg/kg group demonstrated only a mild reduction in white pulp size, with the overall splenic structure largely preserved. In the left foot tissue, substantial lymphocyte and granulocyte infiltration was observed in the 5 mg/kg group (Fig. 7C, blue arrows), while only minimal lymphocytic infiltration was detected in the 10 mg/kg group. In the skin, the 5 mg/kg group exhibited extensive collagen fiber necrosis and lysis (Fig. 7D, orange arrows), accompanied by diffuse infiltration of lymphocytes, granulocytes, and macrophages (Fig. 7D, blue arrows), as well as severe edema and loosening (Fig. 7D, purple arrows) of connective tissue architecture. Conversely, the 10 mg/kg group demonstrated focal infiltration of mast cells and macrophages, accompanied by unremarkable muscle fiber morphology and well-organized tissue structure. These findings were consistent with the quantitative histopathological damage scores (Fig. 7E). Collectively, these results confirm that a 10 mg/kg dose of 4′-FlU effectively suppresses CHIKV replication in mice and significantly reduces pathological damage to the affected target organs.

## DISCUSSION

Chikungunya fever is disseminating globally, thereby posing a substantial public health challenge. Current prevention strategies prioritize mosquito control, case surveillance, and symptomatic treatment. However, there is a paucity of specific medications or vaccines available (1). This highlights the urgent need for the screening of antiviral drugs and the development of vaccines. Conducting high-throughput drug activity screenings against CHIKV is imperative for the advancement of treatment options. While CHIKV has a moderate individual risk and is transmitted by mosquitoes and aerosols, the lack of specific treatments necessitates strict safety measures in Biosafety Level 3 laboratories, impeding progress in drug development. The present study employed VLA1553, the inaugural FDA-approved live vaccine strain, to engineer a replication-competent Nluc reporter virus. This particular strain has demonstrated notable genetic stability, thereby facilitating the real-time observation of viral infection dynamics in murine models. Consequently, it promotes the advancement of research in Biosafety Level 2 facilities.

The insertion of reporter genes into specific genomic locations using reverse genetics techniques is crucial for determining whether a reporter virus can accurately replicate, exhibit pathogenic properties, and maintain the stability of the parental virus. Although several fluorescent- and luciferase-based CHIKV reporter viruses have been reported (Table S3), their stability and application scope are strongly influenced by the reporter insertion strategy (24–34). The construction of CHIKV reporter viruses generally involves the insertion of an additional subgenomic promoter at the junction between the nonstructural and structural proteins. This insertion has been demonstrated to drive the expression of the reporter gene (15, 25). However, these constructs frequently exhibit suboptimal stability. This instability is primarily attributable to the transcription of the inserted reporter gene from an independent subgenomic RNA, thereby compromising its reliability as a screening marker. Furthermore, homologous recombination with subgenomic promoters frequently results in the deletion of the reporter gene. A currently recognized method to enhance stability involves fusing the reporter gene to the less conserved C-terminal region of the nsP3 protein (14). This approach facilitates the concurrent monitoring of the translation of the newly introduced genomic RNA and the translation products of the full-length progeny genome. In this study, the Nluc reporter gene was introduced at amino acid position 490 of the nsP3 protein to create a fusion protein. Consequently, a reporter virus for CHIKV was successfully constructed, as previously reported. The present study’s findings demonstrated that the replication kinetics, the capacity to induce paw swelling in mice, and the lethal pathogenicity of the reporter virus closely resembled those of the wild-type strain. Furthermore, it was observed that stable Nluc fluorescence activity attained levels of up to 10⁷ and persisted after 10 successive passages. This study has not yet assessed the long-term *in vivo* genetic stability of the CHIKV-ΔnsP3-Nluc strain. After the infection of type I interferon-deficient A6 mice with this particular strain, the mice exhibited a median survival duration of 3–4 days (Fig. 3E). The temporal limitation precluded the assessment of Nluc stability in tissues 7–14 days post-infection. However, an analysis of the Nluc activity of this reporter strain in A6 mice between 1 and 3 days after infection was conducted (Fig. 3 and 4). The results indicated that Nluc fluorescence activity remained relatively stable both systemically (Fig. 3G and I) and in various susceptible tissues (Fig. 4B), suggesting that the Nluc reporter gene in this strain demonstrates good early stability in A6 mice. These findings suggest that the constructed reporter strain effectively mimics the biological characteristics of wild-type CHIKV in both *in vivo* and *in vitro* contexts. Compared with previously reported reporter CHIKV systems, CHIKV-ΔnsP3-Nluc was constructed on an attenuated VLA1553-related ΔnsP3 backbone and used an nsP3-based Nluc insertion strategy. This design combines an attenuated vaccine-derived backbone with stable reporter expression, supporting its application in antiviral screening, *in vivo* imaging, and antiviral efficacy evaluation (Table S3).

The present study did not utilize immunocompetent wild-type mice, such as C57BL/6 or BALB/c, given that the CHIKV-ΔnsP3-Nluc strain is an attenuated vaccine with reduced replication capacity. According to the extant literature, following infection with CHIKV, wild-type mice exhibit short-lived viral persistence, do not develop severe symptoms, and rarely experience weight loss (35–37). Consequently, the immunodeficient mouse model (Ifnar1⁻/⁻) was selected for antiviral drug screening due to its enhanced genetic purity and experimental stability, rendering it suitable for investigating Flaviviridae and Enveloped Viruses that depend on type I interferon-mediated immunity (38). In this study, the CHIKV-ΔnsP3-Nluc virus was utilized for real-time monitoring of CHIKV infection in A6 mice through *in vivo* imaging. This methodology enabled the observation of viral propagation from the injection site to various abdominal organs without the need to sacrifice the mice. A robust correlation was identified between bioluminescence activity and viral replication levels, facilitating concurrent monitoring of body weight, survival curves, and viral replication. The study’s findings indicated that CHIKV-ΔnsP3-Nluc predominantly disseminated to the abdominal cavity, with the highest viral titers detected in the spleen and muscle tissues. In type I interferon-deficient mice, it has been demonstrated that CHIKV can infect nearly all tissues, including the heart and central nervous system. However, the CHIKV-ΔnsP3-Nluc virus exhibited minimal infection in these areas, as indicated by low NLuc fluorescence. Despite the presence of elevated viral titers in brain and heart tissues from A6 mice infected with CHIKV-ΔnsP3-Nluc (Fig. S2A), NLuc activity was found to be significantly lower in comparison to organs such as the liver and spleen (Fig. S2B; Fig. 4B). This observation suggests that the low fluorescence levels may be attributable to the inefficient penetration of the luciferase substrate, Nano-Glo Fluorofurimazine, into these particular tissues.

Recent studies have confirmed that the nucleotide analog 4′-FlU is a potent inhibitor of CHIKV replication (22). In mouse models, oral administration of 4′-FlU has been demonstrated to markedly decrease viral tissue load and ameliorate symptoms induced by the virus (39). Furthermore, 4′-FlU has been shown to potently impede the replication of numerous RNA viruses, including SARS-CoV-2, respiratory syncytial virus, influenza virus, Onyong-nyong virus (ONNV), and Mayaro virus (MAYV) (22, 40, 41). The mechanism of action of this compound involves targeting the non-structural protein 4 (nsP4)-dependent RNA polymerase of the virus, leading to the premature termination of RNA synthesis through its incorporation into the growing RNA chain (22, 39). This process culminates in a broad-spectrum antiviral effect. In this study, 4′-FlU was selected as a positive control drug against CHIKV. The *in vivo* efficacy evaluation in mice included three oral dose gradients of 5 mg/kg, 10 mg/kg, and 20 mg/kg. The monitoring of body weight revealed a substantial decrease in weight within the 20 mg/kg cohort over a period of 7 days (Fig. S3). This observation suggests the presence of pronounced toxicity at this 20 mg/kg dosage. Consequently, subsequent comparisons concentrated on the protective effects of 5 mg/kg versus 10 mg/kg against CHIKV-ΔnsP3-Nluc infection in mice. The findings of the present study demonstrated that both doses were effective in reducing viral tissue load and delaying paw swelling in mice. However, mortality was observed in the 5 mg/kg group by day 7 post-infection. In contrast, no deaths occurred in the 10 mg/kg group, indicating that the 10 mg/kg dose provides superior protection against CHIKV infection. It is imperative to acknowledge the inherent complexity in determining the optimal effective dose of 4′-FlU for *in vivo* applications based solely on these two dose gradients. Subsequent systematic dose-response studies are required to evaluate its safety and efficacy in animal models comprehensively.

In addition, to further verify whether the reporter strain developed in this study could be used for screening CHIKV neutralizing antibodies, neutralization assays were conducted using serum from mice in the recovery phase of CHIKV infection (Fig. S6A). The results demonstrated that convalescent serum exhibited a substantial inhibitory effect on CHIKV-ΔnsP3-Nluc infection, even at low dilutions. As the dilution level increased, the neutralizing titer decreased in a stepwise manner. Furthermore, the Nluc activity exhibited a strong correlation with the RT-qPCR results, demonstrating a typical dilution-dependent neutralization pattern (Fig. S6B and C). This finding suggests that Nluc activity may serve as a reliable indicator of the serum’s neutralizing effect against CHIKV infection.

VLA1553 is the first live-attenuated vaccine for CHIKV to receive full FDA approval. Launched in 2023, it is currently approved in a limited number of countries. Notably, CHIKV-ΔnsP3-Nluc, a construct derived from the vaccine strain, shows reduced virulence and significantly lower pathogenicity. However, experimental activities involving the cultivation, amplification, and infection of cells with this strain in BSL-2 laboratories are still subject to biosafety risk assessments and administrative approval. Its implementation depends on obtaining confirmation and approval, which are prerequisites for its use in BSL-2 laboratories.

In summary, the present study successfully developed a genetically stable replicating CHIKV reporter virus, designated CHIKV-ΔnsP3-Nluc, using an attenuated vaccine strain of CHIKV. This novel reporter virus facilitates *in vitro* drug screening and *in vivo* imaging in Biosafety Level 2 laboratories. It elucidates the replication dynamics of CHIKV in cells and mice, confirming its utility for characterizing viral replication and pathogenic mechanisms. The platform’s efficacy in drug screening and safety assessments underscores its potential for developing anti-CHIKV therapies and expediting clinical treatment options.

## Data Availability

The data that support the findings of this study are available within the article and its supplemental material.

## SUPPLEMENTAL MATERIAL

Supplemental material jvi.00466-26-s0001.docx Fig. S1 to S6; Tables S1 to S3.

ASM does not own the copyrights to Supplemental Material that may be linked to, or accessed through, an article. The authors have granted ASM a non-exclusive, world-wide license to publish the Supplemental Material files. Please contact the corresponding author directly for reuse.
