# Supplementary material for: A nanoluciferase-expressing attenuated CHIKV vaccine strain enables rapid and visual drug screening in vitro and in vivo
Source: J Virol. 2026 Aug 11;100(9):e00466-26. doi: 10.1128/jvi.00466-26 (PMC13595972; doi:10.1128/jvi.00466-26)
Supplement: Supplementary file 1 [file jvi.00466-26-s0001.docx]

**Supplemental Figure 1.** Schematic representation of the construction of the genomic-length CHIKV-ΔnsP3 and CHIKV-ΔnsP3-Nluc cDNA clones. Strategy for constructing the infectious cDNA clone of the reporter virus.

**Supplemental Figure 2.** Replication and dissemination of CHIKV-ΔnsP3-Nluc in A6 mice heart and brain tissues. (A) Viral titers in the heart and brain of mice infected with 10^4^ PFU of CHIKV-ΔnsP3, 10^3^ PFU of CHIKV-ΔnsP3-Nluc, and 10^4^ PFU of CHIKV-ΔnsP3-Nluc. Mice were euthanized at 1, 2, and 3 d.p.i. (n = 3; error bars represent standard deviation) (B) The replication of CHIKV-ΔnsP3-Nluc in the heart and brain of each mouse as determined by quantification of bioluminescence signal, calculated as photon ﬂux (photons/s) at 1 d.p.i., 2 d.p.i., and 3 d.p.i., corresponding to (Fig.4A)

**Supplemental Figure 3.** Changes in body weight of mice treated with various doses of 4'-FlU. Mice were given either a vehicle control or 4'-FlU at doses of 5, 10, or 20 mg/kg once daily for 7 days. Body weight was measured daily, with the initial body weight set at 100% (n = 3 per group). Data are presented as mean ± standard deviation (SD).

**Supplemental Figure 4.** 4'-FlU reduced the histopathological changes observed in various tissues of A6 mice infected with CHIKV-ΔnsP3-Nluc on the third day post-infection. The representative images display H&E staining of the spleen from mice infected with CHIKV-ΔnsP3-Nluc, which were treated with either a vehicle at 5 mg/kg or with 10 mg/kg of 4'-FlU after three days. These images are compared to those of mock-infected mice. The scale bars represent 500 μm for the lower-magnification images (×5), and boxes in these images indicate areas where higher-magnification images (×20) were captured. Additionally, representative isolated organs, including the kidney, left muscle, right muscle, left foot, right foot, and skin, are shown.

**Supplemental Figure 5.** 4'-FlU alleviated the histopathological changes observed in various tissues of A6 mice infected with CHIKV-ΔnsP3-Nluc on the seventh day post-infection. The representative images show H&E staining of the spleen from mice infected with CHIKV-ΔnsP3-Nluc and treated with either a vehicle, 5 mg/kg, or 10 mg/kg of 4'-FlU after three days, compared to mock-infected mice. Scale bars represent 500 μm for lower-magnification images (×5), while the boxes in these images indicate the areas where higher-magnification images (×20) were taken. The figure also includes representative images of isolated organs, including the kidney, left muscle, right muscle, and right foot.

**Supplemental Figure 6.** Efficacy of CHIKV-ΔnsP3-Nluc in a Neutralization Assay Using Serum from Mice During the Recovery Phase of CHIKV Infection. (A) Schematic of experimental design.C57BL/6 mice were intradermally injected with 10^6^ PFU of CHIKV-ΔnsP3. Collect serum 28 days after infection. (B) Diluted sera effects on CHIKV-ΔnsP3-Nluc replication in Vero cells as determined by qRT-PCR. (n = 3, error bars indicate SD from triplicates). (C) Diluted sera effects on CHIKV-ΔnsP3-Nluc replication in Vero cells. Luciferase activities were measured at 48 h.p.i. (n = 3, error bars indicate SD from triplicates).

**Supplemental Table 1.** Primers Used in PCR for Constructing the Reporter Virus.

| **Primers** | **Name** | **Sequence (5’-3’)** |
| --- | --- | --- |
| Primer 1 | LR-PacI-T7-F | ctgctgacgCCTTAATTAAGGTAATACGACTCACTATAGATGGCTGCG |
| Primer 2 | LR-nsp1-3-R | GCCGCAAGGTTTCCCCTTGGACTTACGCGCGATGGC |
| Primer 3 | LR-nsp4-sp-F | CGTAAGTCCAAGGGGAAACCTTGCGGCCGTGTCTGATTGGG |
| Primer 4 | LR-NotI-R | aaggctgcaccggtgTGCGGCCGCATTTTT |
| Primer 5 | LR-Δ-nsp1-3-R | GAGTGTGAAGACCATCAAGCTTTCGATTTCTCCTTCGTTG |
| Primer 6 | NLuc-F | GAAATCGAAAGCTTGATGGTCTTCACACTCGAAGATTTCGTTG |
| Primer 7 | Nluc-R | GAAAGTTAGTAGCTCAGAAGACGCCAGAATGCGTTCGCAC |
| Primer 8 | LR-nsp4-C-F | CATTCTGGCGTCTTCTGAGCTACTAACTTTCGGAGACTTC |
| Primer 9 | LR-nsp4-C-R | CCCTCGGGGGTGATTTTAGTGACAATGTCTTTATTCCAGG |
| Primer 10 | LR-sp-F | GACATTGTCACTAAAATCACCCCCGAGGG |

**Supplemental Table 2.** Weighting Scheme Used for Histopathological Scoring of Skin, Paw, and Spleen Tissues

| **Tissue** | **Lesion** | **Weight** |
| --- | --- | --- |
| Skin | Necrosis | 0.4 |
|  | Inflammatory cell infiltration | 0.3 |
|  | Edema | 0.2 |
|  | Epidermal thickening | 0.1 |
| Paw | Necrosis | 0.4 |
|  | Inflammatory cell infiltration | 0.3 |
|  | Connective tissue hyperplasia | 0.2 |
|  | Edema | 0.1 |
| Spleen | Reduction of white pulp | 0.4 |
|  | Inflammatory cell infiltration | 0.3 |
|  | Extramedullary hematopoiesis | 0.2 |
|  | Increased megakaryocytes | 0.1 |

**Supplemental Table 3.** Characteristics and Applications of Reported CHIKV Reporter Viruses

| **Reporter** | **Viral strain** | **Reporter insertion site** | **Attenuation compared with WT virus** | **Genetic stability** | **In vitro applications** | **In vivo applications** | **Reference** |
| --- | --- | --- | --- | --- | --- | --- | --- |
| GFP | 181/25  (GenBank accession no. MW473668) | Between nsP4 and Capsid (dual subgenomic promoter(SG)) | No significant difference | Stable up to passage 6 | High-throughput antiviral screening | Not evaluated | (24) |
| GFP | LR2006 OPY1  (GenBank accession no. DQ443544) | Dual SG: 5′-GFP (upstream of structural genes) or 3′-GFP (genome terminus) | Attenuated in mosquito cells, similar infectivity in mammalian cells | 5′-GFP relatively stable (50% expression after 10 passages in BHK-21 cells); 3′-GFP unstable (5% expression after 10 passages) | Stability evaluation; comparison of fluorescent and CPE titers | Mosquito infection studies; monitoring GFP expression in midgut and salivary glands | (25) |
| eGFP | CHIKV-JC2012  (GenBank accession no. KC488650) | Between nsP4 and Capsid (dual SG) or upstream of Capsid via 2A peptide | Approximately 10-fold reduction in viral titer | Dual-SG construct stable to P5; 2A construct unstable | Neutralization assay; antiviral screening | Not evaluated | (15) |
| eGFP | 37997  (GenBank accession no. AY726732) | 5′ insertion: downstream of nsP4 and upstream of structural genes; 3′ insertion: downstream of structural genes with an additional SG promoter | Slightly lower bloodmeal titers, but infection rate remained 100%; dissemination efficiency higher in the 5′ construct | Not evaluated | Growth kinetics in Vero, C6/36, and MOS-55 cells | Oral infection of *Aedes aegypti*; visualization of GFP expression in midgut, salivary glands, and neural tissues | (26) |
| mKate2 | 181/25 | Between Capsid and E3 under SG promoter control | Not compared | Not evaluated | Studies of virion assembly | Not evaluated | (27) |
| iRFP | - | Between nsP4 and Capsid (dual SG) | Mild attenuation | Reporter gene loss observed after passage 5 | Correlation between fluorescence intensity and viral titer | Age-dependent virulence studies; real-time imaging of viral dissemination and bone marrow tropism | (13) |
| mCherry | IND/2010/DEL/01  (GenBank accession no. PQ673866) | Between nsP4 and Capsid (dual SG) | Similar to parental virus | Stable for five alternating passages | Viral characterization and serum neutralization assays | Not evaluated | (28) |
| mCherry | 37997 | Between E3 and E2 | Markedly attenuated | Stable | Cryo-EM structural studies and protein analysis | Lethal, chronic, and immunological mouse models | (29) |
| mCherry | Mauritius strain  (GenBank accession no. FJ959103) | Inserted within nsP3 (aa377 or aa490) | Similar to parental virus | Stable for at least four passages | Replication complex localization and growth kinetics studies | Not evaluated | (14) |
| hRluc | LR2006-OPY1 | Between nsP4 and Capsid (dual SG) | Similar to parental virus | Not tested | Luciferase imaging in Vero and C6/36 cells at 24 hpi | In vivo imaging in mice and mosquitoes to monitor viral dissemination | (30) |
| NanoLuc | LR2006-OPY1 | C-terminus of nsP3 | Not compared | Not tested | Antiviral mechanism studies | Not evaluated | (31) |
| NanoLuc | BNI-CHIKV 899  (GenBank accession no. FJ959103) | Between nsP3 and nsP4 | Similar to parental virus | Stable over 10 passages | Infection of primary chondrocytes and mechanistic studies | In vivo imaging and cartilage tropism studies in mice | (32) |
| NanoLuc | 181/25 | Downstream of the furin cleavage site between E3 and E2 | Mild attenuation with smaller plaque size | Not tested | High-throughput screening and mechanistic studies | Not evaluated | (33) |
| NanoLuc | 181/25 | Downstream of Capsid (between Capsid and E2) | Similar to parental virus | Not tested | High-throughput screening; dose-response and time-course antiviral studies | Not evaluated | (34) |
| NanoLuc | LR2006-OPY1  Within nsP3 hypervariable region (183-nt deletion at aa490 site) | nsP3 aa490 | Similar to parental virus | Stable over 10 passages | Drug screening and neutralization assays | In vivo drug screening and bioluminescence imaging in mice | This study |
